# Anaemia in Sheep Caused by *Babesia* and *Theileria* Haemoparasites

**DOI:** 10.3390/ani12233341

**Published:** 2022-11-29

**Authors:** Sergio Villanueva-Saz, Marta Borobia, Antonio Fernández, Calasanz Jiménez, Andrés Yzuel, María Teresa Verde, María Ángeles Ramo, Luis Figueras, Héctor Ruíz

**Affiliations:** Animal Pathology Department, Instituto Agroalimentario de Aragón-IA2 (Universidad de Zaragoza-CITA), Facultad de Veterinaria, C/Miguel Servet 177, 50013 Zaragoza, Spain

**Keywords:** *Babesia*, lamb, *Theileria*, sheep, anaemia

## Abstract

**Simple Summary:**

Vector-borne diseases are a group of diseases caused by different pathogens, including viruses, bacteria or parasites transmitted by the bite of hematophagous arthropods, and have a direct impact on livestock, especially animals exposed to vector bites. Among these vector-borne diseases, *Babesia* and *Theileria* in sheep cause anaemia. *Theileria* species can infect two types of blood cells, including leukocytes and erythrocytes, while *Babesia* species infect only erythrocytes. Clinical signs vary in severity from subclinical infection to severe clinical picture with anaemia, depending on different factors such as the immune response developed by the host against the parasite, the parasite load, and the haemoparasites species involved, among others. This review aims to explain the main clinicopathological findings related to these two vector-borne pathogens in sheep, emphasising the pathogenic mechanisms associated with anaemia.

**Abstract:**

Piroplasmoses in sheep are caused by vector-borne apicomplexan protozoa, *Babesia* and *Theileria*. Different species are responsible for the disease; some species are more pathogenic than others and have a worldwide distribution. In this sense, these causative agents can cause anaemia in flocks. In general, these vector-borne diseases infect small ruminants and cause host-mediated pathology. In the case of *Babesia* species, a combination of different mechanisms is involved: red blood cell lysis due to intracellular parasite multiplication, activation of biogenic amines and the coagulation system with the possibility of disseminated intravascular coagulation. By contrast, less information is available on the different immunopathogenic mechanisms involved in the development of anaemia in sheep with theileriosis. However, the mechanisms of pathogenic action in theileriosis are similar to those studied in babesiosis. Diagnosis is based on compatible clinical signs, laboratory findings, specific diagnostic tests and the presence of the tick vector. Some of these tests detect the causative agent itself, such as direct identification by light microscopy and molecular analysis. In contrast, other tests detect the sheep’s immune response to the organism by serology. Both diseases pose a significant diagnostic challenge for veterinary practitioners around the world. This review presents the most frequent clinical signs, pathogenesis and clinicopathological findings, diagnosis.

## 1. Introduction

Vector-borne diseases are caused by different groups of pathogens, including bacteria, parasites and viruses transmitted by the bite of arthropods when they feed on the blood of the host. Among these pathogens, some are classified as haemoparasites and are characterised by the invasion of the bloodstream [1].

This group of diseases has been experiencing an expansion during the last few years. This situation may be favoured by several factors, such as globalisation, migratory animal movements, climate change and modifications in the distribution and location of the vectors involved in the transmission. Due to this reason, the diagnosis and control of these diseases should be approached by a multifactorial perspective based on the “One Health” concept [2,3,4].

In general, in these vector-borne diseases, the diagnosis of the infection is essential. The combination of different diagnostic techniques to confirm the condition allows a concrete overview of the epidemiological situation of the infection produced by one of these pathogens. Using a single method can lead to an underestimation of the actual prevalence of infection. Each confirmatory technique gives precise information about a particular aspect, and combining the results allows a global knowledge of the epidemiological setting studied to be obtained.

*Babesia* and *Theileria* species are tick-borne haemoprotozoan parasites that infect livestock in different regions, including tropical and subtropical areas. Traditionally, piroplams have been included under the name of “piroplasm” due to the pear shape of the parasites after multiplication, resulting in these two diseases named “piroplasmoses” [5].

The classical taxonomy of piroplasms comprises *Babesia* and *Theileria* species. Before the application of molecular techniques, taxonomic classification was focused on morphological and biological characteristics, including three different groups of piroplasmids: *Babesia* sensu strictu with the absence of schizonts and transovarial transmission, *Theileria* with the presence of schizonts and transstadial transmission, and finally *Babesia* sensu latu characterised by piroplasmids that can not be classified as *Babesia* sensu strictu or *Theileria* [6].

The life cycle of the parasites is related to an intermediate host and a definitive host, a tick. In babesiosis, ticks become infected following the ingestion of erythrocytes infected with merozoites. The sexual reproduction of the *Babesia* parasites in the tick gut (gametogony) is followed by an asexual process (sporogony) in its tissues. After that, the formation of the infective stages (sporozoites) occurs in the salivary glands. Two different routes of transmission are described in the life cycle of *Babesia* parasites, including transstadial transmission from one stage of the tick’s life cycle to the next and transovarial transmission, transmitting *Babesia* parasites from female adult tick to their eggs [6].

The life cycle of *Theileria* species is similar to *Babesia*, with a sexual phase (gametogony) followed by asexual reproduction (sporogony) in the tick gut. Finally, an asexual phase occurs in the sheep. It is important to remark that only transstadial transmission has been confirmed in *Theileria* spp., and no evidence of transovarial transmission has been detected [6]. Another difference from the *Babesia* lifecycle is that sporozoites are located only in salivary glands and initially infect host leukocytes. Then sprozoites multiply after a short time, resulting in infectious merozoites that are able to infect other erythrocytes.

Piroplasmoses result in substantial economic costs worldwide due to reduced meat and milk production of infected animals [7]. In addition, the economic impact is even higher as a consequence of the direct mortality and treatment costs [8,9]. The presence of asymptomatic carrier animals may promote the spread of these diseases in areas traditionally considered not endemic in conjunction with the international trade of animals and the presence of competent vectors to transmit the infection [10].

From a clinical point of view, anaemia is the most common clinical sign associated with these haemoprotozoan parasites. Anaemia is defined as an abnormal condition characterised by the decrease of the haematocrit (Packed Cell Volume, PCV), the mass of erythrocytes (Red Blood Cells, RBCs) and/or haemoglobin [11]. Three different mechanisms of anaemia production are described: haemorrhagic, haemolytic and anaemia caused by decreased production of erythrocytes. The first two mechanisms are considered regenerative anaemia, while the third type is classified as non-regenerative anaemia [11,12]. Several causes have been associated with haemolytic anaemia, with haemoparasites being one of the most important causes included in this group [11].

This review is focused on *Babesia* and *Theileria* species in sheep in Europe, including the most relevant aspects of epidemiology, pathogenesis, clinical features, diagnosis, treatment, and prevention of these infections.

## 2. Babesia 

### 2.1. Epidemiology and Transmission

The first case affecting cattle was reported from Romania in 1888 by Dr Victor Babes, a Romanian physician [13]. It was referred to as bovine hemoglobinuria or red water fever in cattle with the presence of microorganisms in erythrocytes. Some years later, the same author described the microorganisms in sheep with haemoglobinuria [14]. In 1893, Starcovici named this pathogen *Babesia ovis* in sheep, while in cattle, two different *Babesia* species were named: *Babesia bovis* and *Babesia bigemina* [15]. Parasites of this group can infect human beings, livestock, pets and wild animals through tick bites [16]. The geographical distribution of *Babesia* infections is highly variable and associated with the distribution of the competent tick vector [17,18] from different genera: *Rhipicephalus*, *Haemaphysalis*, and *Hyalomma* [19,20,21,22,23].

Babesiosis in small ruminants may be caused by several species, *Babesia ovis* being the most prevalent species with a worlwide distribution [24,25,26], followed by *B. motasi* and *B. crassa* [17]. However, there could be differences in prevalence caused by the geographical distribution of each *Babesia* species and vector species. Differences in pathogenicity have also been observed in the same *Babesia* species. *B. motasi* comprises at least two species or subspecies with variable pathogenicity: high pathogenicity in Mediterranean Basin and low pathogenicity in Northern Europe. Other uncommon species, such as *B. crassa* are able to cause infection in sheep, but they do not appear to generate obvious clinical signs in infected animals [27]. There is some evidence of other species, including *B.* sp. *Xinjian* and *B.* sp. *BQ1*, described in China as novel *Babesia* species [28,29,30]. Finally, other common *Babesia* species associated with haemolytic disease have been detected in sheep, such as *Babesia lengau*-like organisms [31].

Some *Babesia* species that infect animal hosts have demonstrated zoonotic potential, being able to infect human beings [32]. However, *Babesia* species that are able to infect sheep do not infect humans, although recently, a case of human babesiosis caused by *B. crassa*-like pathogen has been reported [33]. In sheep, some bovine *Babesia* species have been detected in blood samples and ticks collected from sheep, although the clinical impact of these findings is not well known [34].

### 2.2. Pathogenesis

This pathogen causes host-mediated disorders. Anaemia is attributed to a combination of intravascular and extravascular haemolysis due to different mechanisms, including red blood cell lysis by intracellular parasite multiplication, increased osmotic fragility of the erythrocytes by oxidative changes and activation of biogenic amines and intravascular haemolysis [35]. Although no other pathophysiological mechanisms have been described in sheep, other similar mechanisms may likely occur in animal species, such as dogs, for which more scientific information is available. A variety of potential pathological mechanisms, including the production of aberrant anti-erythrocyte membrane antibodies, is the cause of immune-mediated haemolytic anaemia [36]. The presence of erytrophagocytosis can be observed in smears from the spleen and liver in sick animals such as dogs [37] and cattle [35]. Sheep have been evaluated as an experimental animal model of bovine babesiosis caused by *B. divergens* as they do not show evident clinical signs but develop a robust humoral response [38]. The spleen has a critical function in controlling clinical disease and experimentally infected splenectomised animals rapidly develop parasitemia and clinical signs [38]. A similar situation is observed in canine babesiosis due to *B. rossi* [39] or *B. canis* [40].

Once infected ticks have fed on sheep blood, the protozoan parasites adhere to the red blood cell membrane, penetrating into the red blood cells. After that, haemoparasites multiplication occurs with the formation of merozoites and red blood cell lysis. Lesions of the blood cell membrane accompany this situation due to cell membrane fragility and intravascular and extravascular haemolysis. After the red blood cell destruction with massive multiplication of the parasites in the cell, new haemoparasites invade new erythrocytes in the bloodstream, continuing the extensive proliferation of the parasite [17]. In some patients with a significant reduction in red blood cells and severe cases of acute babesiosis, hypoxia may occur in organs and tissues. It may cause clinical manifestations associated with the affected tissues. In some circumstances, the possibility of disseminated intravascular coagulation syndrome and the lesion of the basal membrane of the glomeruli is described in the acute form of sheep babesiosis [41]. Autoimmune anaemia has been described as associated with babesiosis in human beings and dogs [42,43]. However, no evidence of this type of anaemia has been detected in ruminants.

Another critical point is the variable information about the relationship between parasite load and severity, considering each *Babesia* species that affects sheep, although, in general, high parasitemia is related to severe clinical signs and laboratory abnormalities [44,45]. However, parasitemia levels could differ depending on the *Babesia* species present; thus, some authors consider some species non-pathogenic [46]. Animals infected for the first time probably develop more severe signs. However, following experimental infection with *B. ovis* in sheep, the parasitic load was kept low, as well as the antibody response and duration of latent infection [47].

### 2.3. Clinicopathological Findings

Clinicopathological findings result from the intracellular invasion of the erythrocytes by *Babesia* species. In this sense, these parasites are able to cause clinical signs in the animal, such as fever, haemoglobinuria, jaundice and anaemia. This last sign occurs in 30–50% of cases [48]. However, clinical manifestations are variable and depend on the *Babesia* species causing infection and its associated pathogenic virulence. In addition, other factors could directly influence the severity of the clinical disease, such as susceptibility, the competence of the immune system, parasite load, presence of concomitant diseases, and immunosuppression status, among others [49] (Table 1).

Clinical manifestations may range from absent to fatal, with mild or moderate presentations being the most common finding. Fatal presentations are related to acute circulatory shock. Sheep with acute babesiosis typically present clinical signs such as fever, jaundice, haemoglobinuria and pale mucous membranes due to anaemia. Other non-specific clinical signs such as lethargy, anorexia, weakness and a poor general condition could be detected during the physical examination due to wasting condition [23].

Two different clinical forms could be observed in animals infected by *B. motasi*. In the case of the acute form in natural conditions, it is possible to detect fever, anorexia, tachypnea, jaundice, hemoglobinuria, diarrhoea, anaemia and finally, death. Under experimental conditions, *B. motasi* infection in sheep is able to cause fever, anorexia, and weight loss [50]. Finally, cough, oedema, and poor condition are described in the chronic form [48].

A recurrent problem in the study of immune response in sheep in natural conditions is the difficulty of assessing immunology and other clinicopathological parameters except for experimental infection [38,47]. In some animals, the massive intracellular parasite multiplication in the red blood cells, together with the presence of haemolytic anaemia, could cause more severe clinical signs, even causing other alterations such as haemostasis, thrombocytopenia and haemoglobinuria [51]. 

Clinical recovery is possible in some animals after clinical disease, but they can act as asymptomatic carriers without evident clinical signs, perpetuating the parasite cycle in nature. These carriers act as infection reservoirs and may infect ticks. However, under certain conditions, the carrier animal can suffer an immunosuppression status leading to the appearance of the clinical signs after parasite multiplication [52]. Inherent resistance/susceptibility to the infection has been associated with particular types of sheep breeds or animals reared in endemic areas. This variability is thought to be the result of an immune response from the host, which may be influenced by the genetic background of the sheep [53,54].

However, there is a description of new *Babesia* species as *Babesia lengau*-like sp. with the presence of anorexia, depression, fever and signs of acute haemolytic disease. Using laboratory findings, it is possible to detect the presence of anaemia and leucocytosis. Red blood cells are characterised by anisocytosis, polychromasia, Heinz and Howell–Holly bodies on blood smears [31].

The most common laboratory alteration is hypochromic microcytic anaemia in sick animals, while in sheep with chronic course disease, it is possible to detect macrocytic and hyperchromic anaemia. There is also a decrease in haemoglobin values and detection of thrombocytopenia in the case of disseminated intravascular coagulation. [45]. Additionally, neutrophilia has been observed under experimental infection by *B. motasi* in sheep [50].

Using laboratory techniques, different effects of *B. ovis* can be detected, such as increased hepatic parameters. An elevation in renal parameters is observed due to the renal damage associated with the glomerulonephritis condition. Hypoalbuminemia and a decrease in total protein are detected due to the hepatopathy problem and urine loss due to renal failure. All these laboratory alterations indicate that the main target organs of lesion involvement are the liver and kidney [41]. Moreover, other biochemical parameters are usually increased, such as unconjugated and conjugated bilirubin, serum glutamic pyruvic acid transaminases, and total serum proteins can be affected by the infection [50].

Serum protein electrophoresis is a crucial biochemical technique used for the investigation of a normal distribution of serum protein fractions (albumin, α-1, α-2, β-1, β-2 and γ fraction) [55]. In small animal veterinary medicine, different serum protein electrophoresis patterns can be detected, from normal patterns to acute-phase protein responses, polyclonal gammopathies, oligoclonal gammopathies or also called restricted polyclonal gammopathies and finally, monoclonal or paraproteinemias [56]. In livestock medicine, it is not a common laboratory technique applied to small ruminants. In small ruminant babesiosis caused by *B. ovis*, differences between goats and sheep are observed [57,58]. Markedly decreased total protein concentrations have also been observed in naturally infected sheep with *B. ovis* [58].

### 2.4. Diagnosis

When investigating vector-borne diseases, combining the data and results obtained from the different clinical procedures, including good case history and a detailed physical examination of the sick animals, is essential. The presumptive diagnosis is based on epidemiological data, the presence of ticks, the clinicopathological findings detected and the positive result obtained by a confirmatory diagnostic technique. In these types of diseases, early diagnosis and successful therapy are the critical steps for its control [24]. A common situation is the existence of carriers with low parasitemia levels. This last circumstance could be a diagnosis challenge when some confirmatory diagnostic techniques are used, such as detecting the parasite by microscope. In-clinic tests, stained (Giemsa, Diff-Quick, modified Wright’s stains) blood smears examination by light microscope is one of the most rapid and cheapest methods for detecting *Babesia* parasites in erythrocytes. In countries where other diagnostic methods are not available, Giemsa staining is considered the gold standard for babesiosis diagnosis in small ruminants [59]. However, the light microscopic examination may not be as sensitive and is also time-consuming in animals with a low parasitemia level due to randomly distributed parasites, especially in carrier animals that include sheep surviving after acute babesiosis with a persistent and chronic infection over time [46].

**Figure 1:**
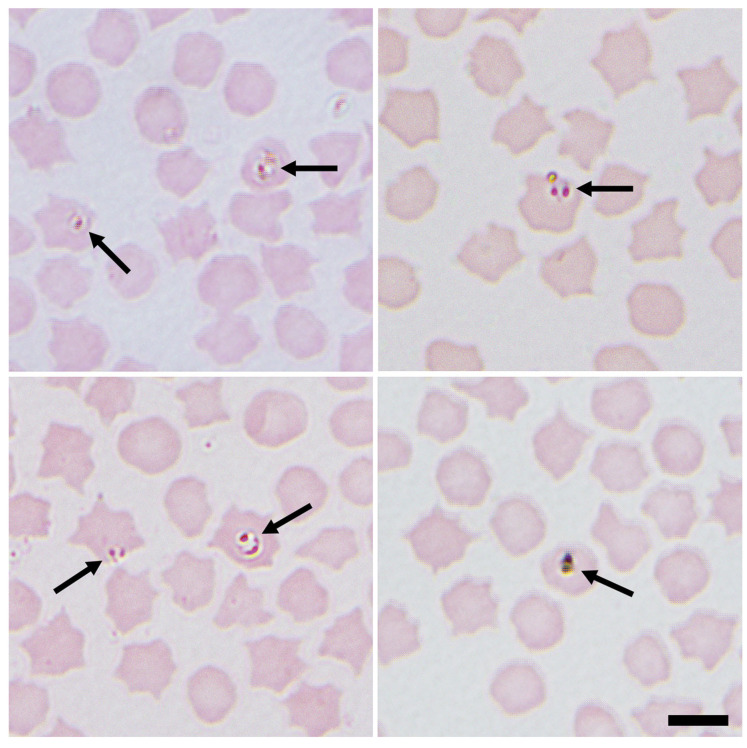
Representative images of small intracellular *Babesia* (arrows) identified in sheep erythrocytes from several sites in northeastern Scotland, UK. Both paired proforms and ring forms are visible. Images were taken at ×1000 magnification with oil immersion. Scale bar indicates 5 μm [60].

**Figure 2:**
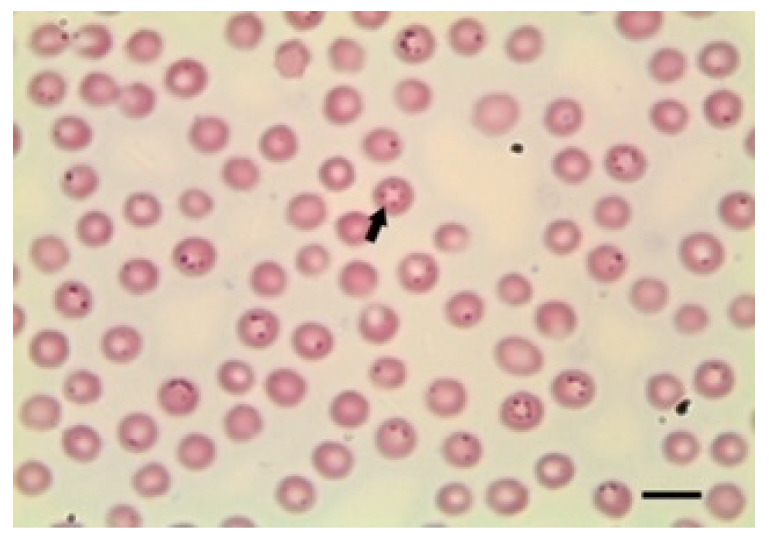
*B. ovis* sporozoites in erythrocytes. Scale bar indicates 5 μm. Image courtesy of Professor Khaled El-Dakhly, Beni-Suef University, Egypt.

**Figure 3:**
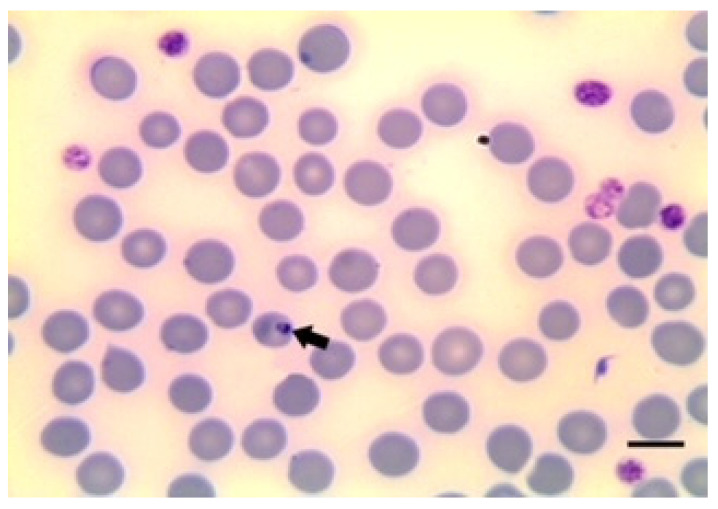
*B. motasi* in erythrocytes. Scale bar indicates 5 μm. Image courtesy of Professor Khaled El-Dakhly, Beni-Suef University, Egypt.

There are descriptions based on morphology of many forms of *Babesia* species. Therefore, this procedure is adequate for classifying the morphology of the parasites in the erythrocytes as either large or small forms by experienced microscopists (Figure 1, Figure 2 and Figure 3).

Small *Babesia* species such as *B. ovis* have a size below 2.5 μm (1–2.5 μm in length) with a round or rare pyriform obtuse angle. These small *Babesia* species are located at the margin of the red cell. There are also large *Babesia* species, such as *B. motasi*, with a size of over 2.5 μm in length. In contrast to the smaller species, in this case, the parasite has a pyriform acute angle [17]. In addition, other species, such as *B. crassa*, have an intermediate size, below 3 μm in length and tetrad forms [61].

To avoid false morphological diagnosis, it is necessary to follow practical recommendations. The maximum magnification of the microscope should be used for correct visualisation of the parasites. The time under the microscope, the number of fields examined and the previous experience of the microscopist in locating and identifying the parasite in the smear are also crucial to making a reliable diagnosis. Artefacts and the quality of blood extension could also interfere with the diagnosis. Therefore, attention to the presence of artefacts and quality of blood extension are necessary.

The second group of diagnostic techniques are serological techniques. This methodology is based on the detection of specific anti-IgG antibodies against *Babesia species* using different techniques, including the enzyme-linked immunosorbent assay (ELISA) and indirect immunofluorescence antibody test (IFAT) [62], these techniques being useful in seroepidemiological studies. 

The main differences between the ELISA and IFAT techniques are the type of antigen used and the method applied to obtain the results. The interpretation of the results varies. IFAT is subjective and depends on the experience of the operator. Even when samples are examined by two experienced observers, however, this situation never arises when the ELISA technique is performed. Various antigens have been used to coat the ELISA plates, such as soluble parasite extracts, culture supernatants or recombinant proteins. Recombinant proteins improve the ELISA test specificity compared with natural parasite extracts [63].

In this type of test, the interpretation of the serological results should be performed with caution. Firstly, a positive result would indicate exposure to parasite infection but does not accurately identify which species are responsible. In addition, a positive serological result can also demonstrate both past or current infection, and seroconversion is not commonly employed in clinical practice. In acute cases of ovine babesiosis, a delay can occur in the antibody production in the sick animals until after the presence of compatible clinical signs, resulting in the possibility of false negative results obtained by serology. Or by contrast, the cross-reaction phenomenon is a common problem among the different *Babesia* species [64]. 

Finally, molecular tests are helpful and highly sensitive in detecting the presence of parasite DNA in a biological sample. These techniques can detect the presence of 10 infected cells in a millilitre of blood sample [65]. Different methods are available, including real-time PCR, nested PCR, conventional PCR or novel techniques such as loop-mediated isothermal amplification technique (LAMP), a rapid molecular test for use in clinical practice without laboratory facilities [66]. However, the routine diagnosis is restricted to specific laboratories. Molecular techniques are also helpful for the identification of the *Babesia* species causing subclinical infections in sheep that do not show apparent clinical signs [52]. Studies have been carried out to identify piroplasmids using different molecular markers, including hypervariable region 4 (V4 region) of the small subunit ribosomal RNA gene [9,67], and the gene encoding the *B. ovis* surface protein D [68].

## 3. Theileria

### 3.1. Epidemiology and Transmission

Theileriosis is another tick-borne infection with high morbidity and mortality. Different species are detected as the causative agent of ovine theileriosis, including *Theileria lestoquardi*, *T. ovis*, *T. recondita*, *T. uilenbergi*, *T. separata* and *T. luwenshuni* [23,69,70,71,72,73]. Considering *Theileria* genotypes, three different types have been identified as *Theileria* sp. OT1, *Theileria* sp. OT3 and *Theileria* sp. MK [69,74,75]. 

In small ruminants, theileriosis is transmitted through the bite of infected ixodid ticks from various genera [23]. However, another transmission route, including transplacental transmission, has been described in the case of *T. lestoquardi* [76]. 

In general, this disease has a relatively limited impact in Europe due to the presence of low pathogenicity species [72]. However, economic losses are detected in other regions of the world. Moreover, there are geographical areas where it is possible to detect the presence of two *Theileria* species and the coinfections of the sheep with the two species [77].

From a clinical point of view, *Theileria lestoquardi* is able to produce clinical disease in sheep in Europe [78], followed by *T. uilenbergi* and *T. luwenshuni* [22,79,80]. By contrast, other species, such as *T. ovis,* cause subclinical infections in immunocompetent animals, while animals under stressful conditions may develop clinical signs. *T. separata* is considered a non-pathogenic species, but in immunocompromised animals can cause fatal situations [81]. *T. recondita* can also be associated with a mildly pathogenic outcomes in specific stress conditions [50].

### 3.2. Pathogenesis

*Theileria* species are able to infect different types of cells from the vertebrate host. In this sense, leukocytes are infected by sporozoites, whilst maturation of schizonts into merozoites occurs in the red blood cells, leading to the formation of piroplasms that are the typical laboratory picture observed in the microscopic examination of blood smears from sick animals [82].

Clinical theileriosis is associated with anaemia due to erythrocyte destruction and haemolysis with lymph node enlargement. In general, the immunopathogenic mechanisms appear similar to those of *Babesia* infection. Depending on the responsible species, clinical differences can be detected. Among the *Theileria* species, *T. lestoquardi* is the most relevant species because these protozoa cause high mortality and morbidity rates [83]. This agent is traditionally associated with a malignant form of ovine theileriosis.

### 3.3. Clinicopathological Findings

In the acute form of *T. lestoquardi*, it is possible to detect the presence of fever, weakness, anorexia, conjunctival petechiae, swollen lymph nodes and jaundice. In chronic forms, non-specific signs such as intermittent fever, inappetence and emaciaton may be observed. The presence of mucosal pallor due to anaemia is also observed in this chronic disease [84]. Other non-specific signs include ruminal hypomotility or cessation of rumination, diarrhoea or constipation, cough, nasal and ocular discharges, increased respiratory rate and tachycardia [85]. In later stages, the presence of other opportunistic infections, such as viruses, bacteria, and fungal infections due to immunosuppression situations, can also be observed [86]. However, several factors such as genetics, nutrition, concomitant infections, infective species or the infective dose may cause different clinical presentations [87].

In the case of *T. ovis*, abnormal respiratory sounds are detected together with mucosal pallor, fever, ruminal hypomotility and anorexia. These signs are more evident in lambs in comparison with adult animals [88]. Infected animals that survive after the acute form become carriers without apparent clinical signs but have fluctuating low parasitemia [89]. When a tick feeds on these carriers, it can transmit to other non-infected animals with a direct impact on livestock [90] (Table 2).

In general, sheep with acute theileriosis typically present anaemia with a decrease in red blood cells and haematocrit due to erythrocyte destruction by the mononuclear phagocyte system [91] and activation of the complement system [92]. In experimental infection of sheep by *T. lestoquardi*, a decrease of the haemoglobin concentration and red blood cells is detected, whilst, in white blood cells, a variable situation of leukopenia/leukocytosis is detected [93].

Laboratory findings include hypoalbuminemia related to hepatic damage, increased liver parameters due to liver damage and cholestatic disorder, increased total protein concentration, and increased blood uremic nitrogen and creatinine levels due to kidney lesions [91,94]. Other laboratory indications are related to the cardiovascular system with electrocardiography alterations and elevation of cardiac troponin I, lactate dehydrogenase, and aspartate aminotransferase caused by *T. lestoquardi* under experimental conditions [95].

### 3.4. Diagnosis

Similar to ovine babesiosis, the combination of results, including epidemiological data, vector presence, host, and *Theileria* species located in the region, are necessary to establish a correct diagnosis [96].

Traditionally, these agents have been diagnosed by low-sensitivity techniques, such as microscopic examination of blood smears. A common problem related to ovine theileriosis is the lack of recommendations from the Office International de Epizooties (OIE) due to it being considered a low or even non-pathogenic species, in contrast to the situation with horses and cattle [69].

**Figure 4:**
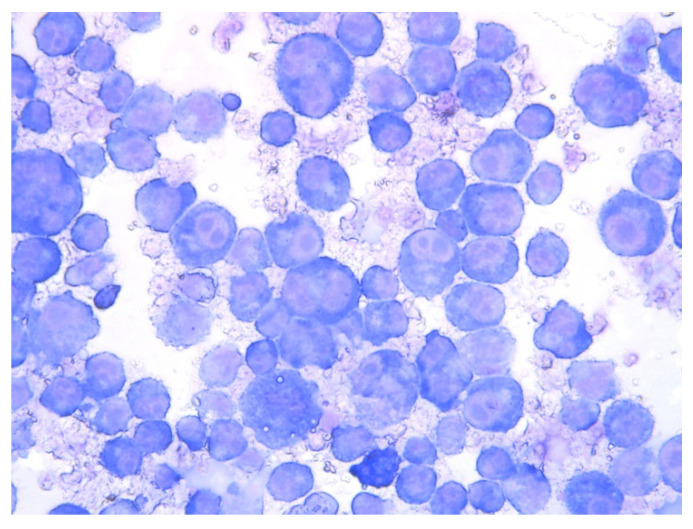
Celular culture with the presence of *T. ovis* schizonts in lymphocytes. Obj. 40×. Biological material courtesy Morocco’s *Pasteur Institute*, *Morocco*.

**Figure 5:**
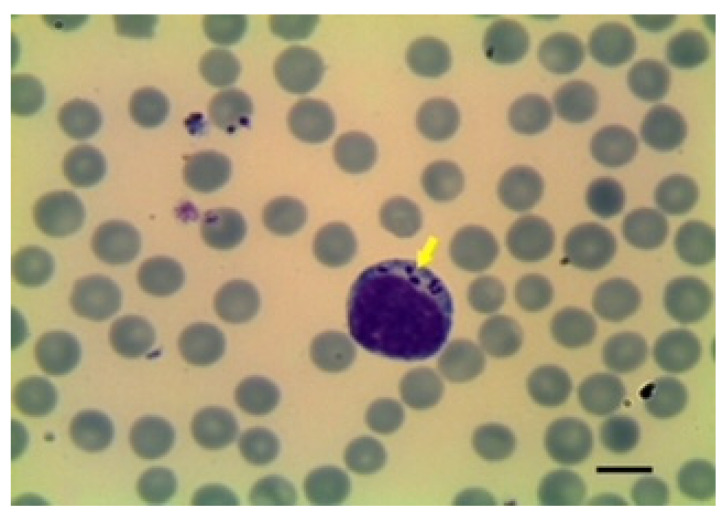
*T. ovis* schizont in blood smear. Image courtesy of Professor Khaled El-Dakhly, Beni-Suef University, Egypt.

Microscopic examination of blood smears is an important diagnostic tool for *Theileria* species. The use of the light microscope was described after Koch’s discovery of *T. parva* [82]. In red blood cells, *Theileria* forms have different shapes (rod, round, oval and ring-shaped) and are up to 2.0 µm long and 1.0 µm wide. In the case of lymphocytes, the schizonts are 8.0 µm in size with a variable number of nuclei [82] (Figure 4 and Figure 5).

In general, this technique has a low sensitivity to detect the presence of pathogens in animals with low parasite load, such as infected carrier animals. Samples from organs other than blood could be used for microscopic examination. These smears could be prepared from other organs, including the liver, spleen, lung, kidney and lymph nodes [97]. Another disadvantage is the time-consuming nature of microscopic examination.

Another negative aspect is the fact that morphological characterisation is subjective and is not always possible. It is dependent on the observer. In the absence of other methods that are highly sensitive and specific, the microscopic examination could be an alternative. However, the use of the light microscope is not a good diagnostic method to detect asymptomatic infected animals that act as carriers in endemic areas.

There are available different serological diagnostic techniques to detect the presence of specific antibodies against the causative agent, such as IFAT [82], ELISA [98] and immunochromatographic strip test [99], with IFAT and ELISA being the most common tests. The difference between these two techniques is the type of antigen used. 

In the case of IFAT, this antigen can be prepared from schizont form obtained from infected sheep or, in other cases, by laboratory culture. By contrast, for ELISA, different types of antigens can be employed, such as crude antigens obtained from lysate merozoite or recombinant protein [100,101], thereby improving the specificity value [102]. For both serological techniques, a laboratory with expensive equipment is necessary to perform the test with trained personnel, but in the case of the ELISA technique, a high number of samples can be screened compared with IFAT. On the other hand, the immunochromatographic test can be used as an important first step in diagnostic algorithms, enabling results to be obtained within a short time [82].

Serological methods are commonly employed in epidemiological surveys, but the possibility of a cross-reaction phenomenon can also occur between related protozoan species [103].

Molecular methods have high sensitivity and specificity compared with other diagnostic techniques, such as microscopic examination and serology [104]. The best samples to perform molecular analysis are blood and spleen [105]. Different molecular techniques are available, including conventional PCR as well as electrophoretic agarose analysis, nested PCR, and other more modern techniques [82,105,106]. The advancement in the molecular field is especially relevant to the differentiation of *Theileria* species that are morphologically identical such as *T. luwenshuni* and *T. uilenbergi* [107]. Recently, new equipment based on LAMP assays has been used under field conditions [108,109]. In general, the common point of these techniques is the detection of parasitic genetic material in sheep at the time of sampling. 

## 4. Conclusions

Ovine babesiosis and theileriosis are two vector-borne diseases with different distributions in Europe. The presumptive diagnosis is based on epidemiological data, the presence of ticks, the clinicopathological findings and positive results obtained by a confirmatory diagnostic technique. Among laboratory findings, anaemia is the most commonly observed. The presence of carriers in the flock is an important aspect of this vector-borne epidemiology which perpetuates the parasite lifecycle in nature. However, early diagnosis and successful therapy are the critical steps for its control. Different types of confirmatory techniques are available; depending on the purpose of pathogen detection, selecting one or other type of confirmatory method is necessary. There are currently no vaccines available to prevent these types of vector-borne diseases, and prevention using different topical antiparasitic drugs against ticks seems to be the best approach. 

## Figures and Tables

**Table 1:** Clinical signs associated with each *Babesia* species.

Species	Presence of Anaemia	Other Clinical Signs	Pathogenicity
*B. ovis*	Yes	Fever Haemoglobunuria Jaundice Wasting condition	++++
*B. motasi*	Yes		
			+++/++
*B. crassa*	No		+

+: level of pathogenicity.

**Table 2:** Clinical signs associated with each *Theileria* species.

Species	Presence of Anaemia	Other Clinical Signs	Pathogenicity
*T. lestoquardi*	Yes	Fever Inappetence Weakness Anorexia Jaundice	++++
*T. uilenbergi*	Yes		+++/++
*T. luwenshuni*	Yes		+++/++
*T. ovis*	No	Subclinical infection	−/+ (immunocompromised animals)
*T. separata*	No	Subclinical infection	−/+ (immunocompromised animals)
*T. recondita*	No	Subclinical infection	−/+ (immunocompromised animals)

”+”: level of pathogenicity; “−”: no pathogenicity.

## Data Availability

Not applicable.
